# Supplementary figures and images for: Structural Adaptation of the Excitation–Contraction Coupling Apparatus in Calsequestrin1-Null Mice during Postnatal Development
Source: Biology (Basel). 2023 Jul 29;12(8):1064. doi: 10.3390/biology12081064 (PMC10452101; doi:10.3390/biology12081064)

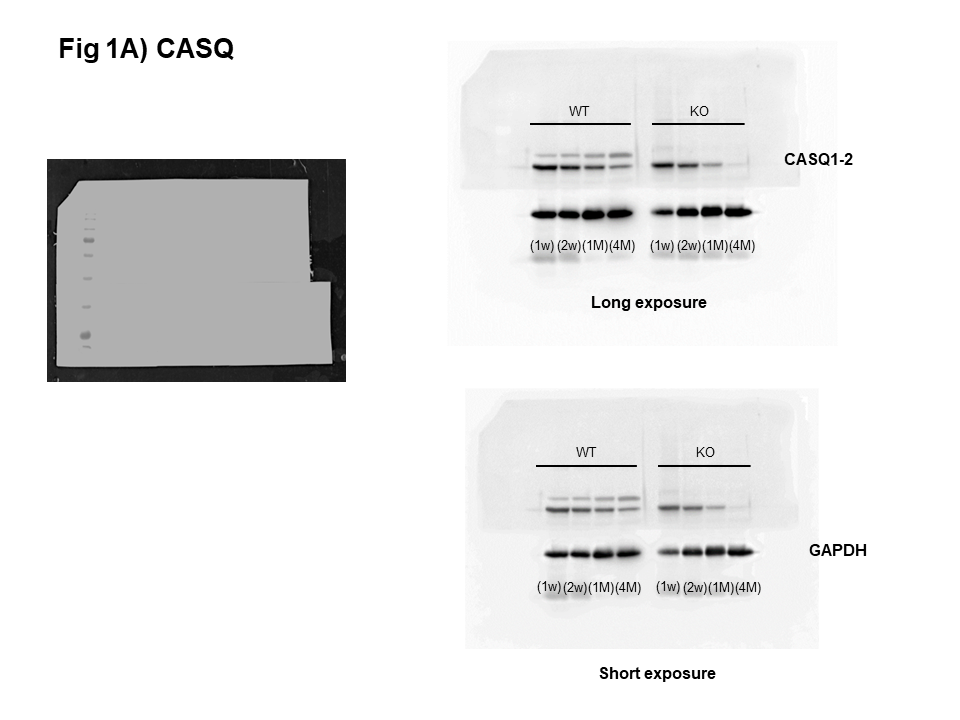

Supplement: Supplementary file 1 [file biology-12-01064-s001.zip › File S1.TIF]

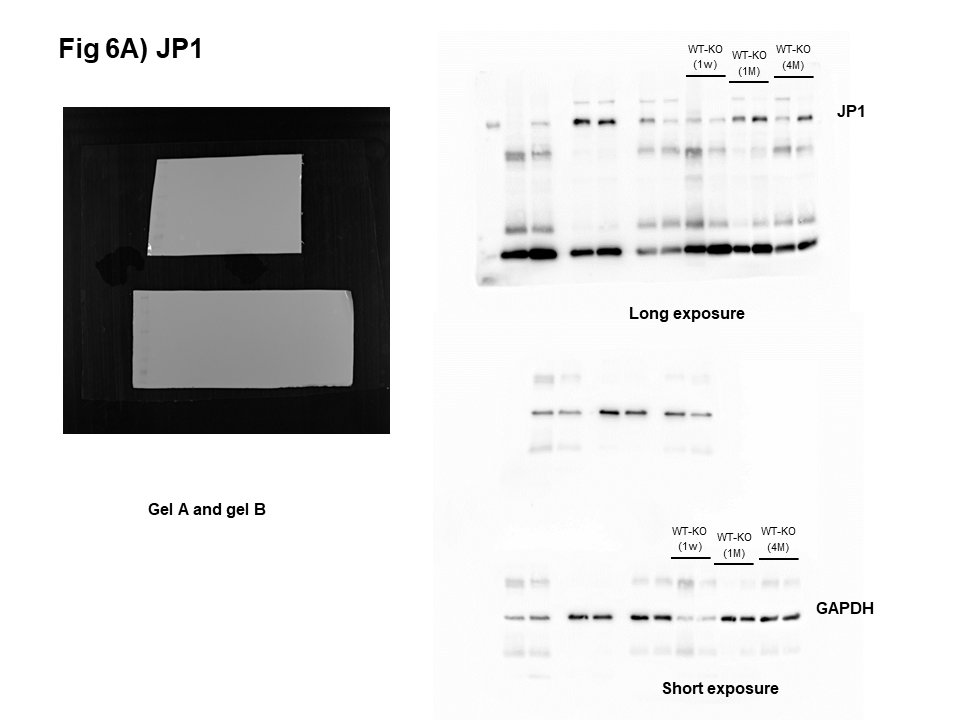

Supplement: Supplementary file 1 [file biology-12-01064-s001.zip › File S2.TIF]

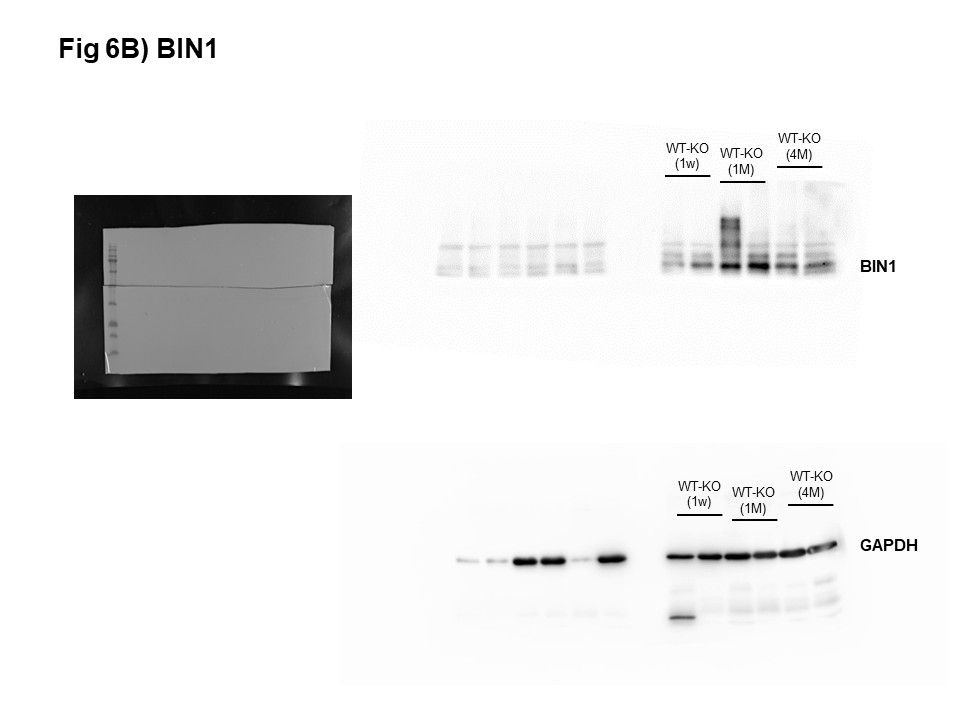

Supplement: Supplementary file 1 [file biology-12-01064-s001.zip › File S3.TIF]

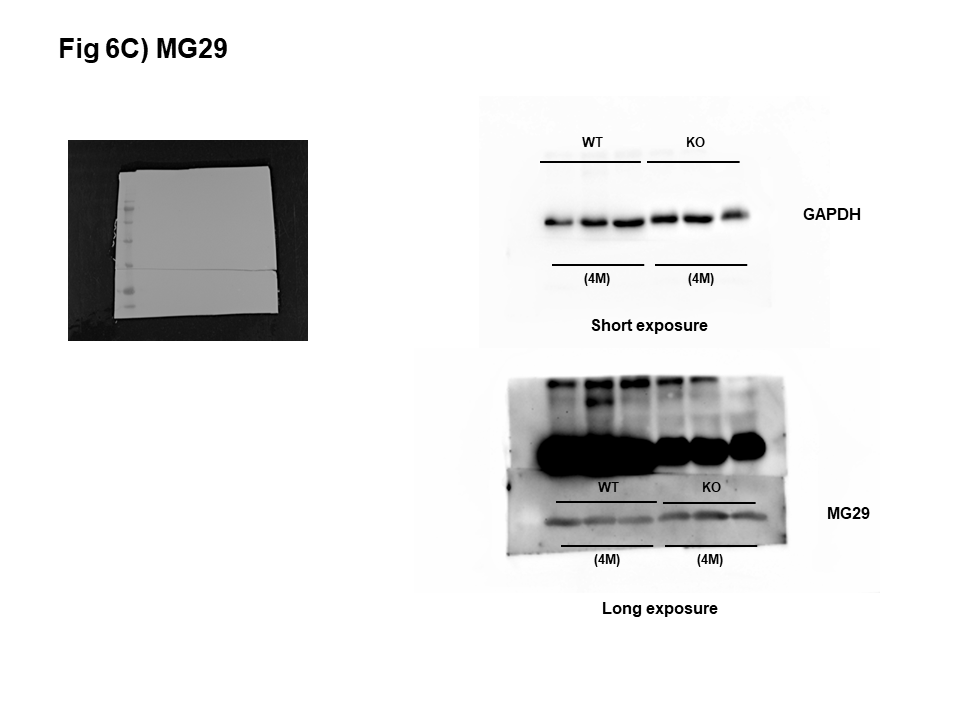

Supplement: Supplementary file 1 [file biology-12-01064-s001.zip › File S4.TIF]

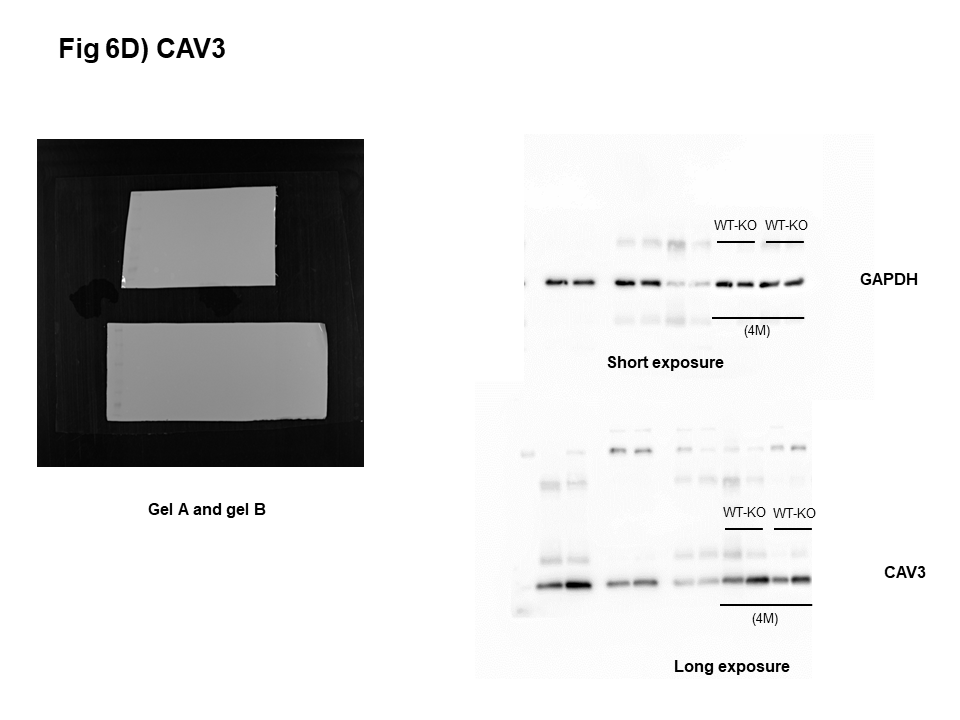

Supplement: Supplementary file 1 [file biology-12-01064-s001.zip › File S5.TIF]
